# Supplementary figures and images for: Dynein and dynactin move long-range but are delivered separately to the axon tip
Source: J Cell Biol. 2024 Feb 26;223(5):e202309084. doi: 10.1083/jcb.202309084 (PMC10896695; doi:10.1083/jcb.202309084)

3SC/E)

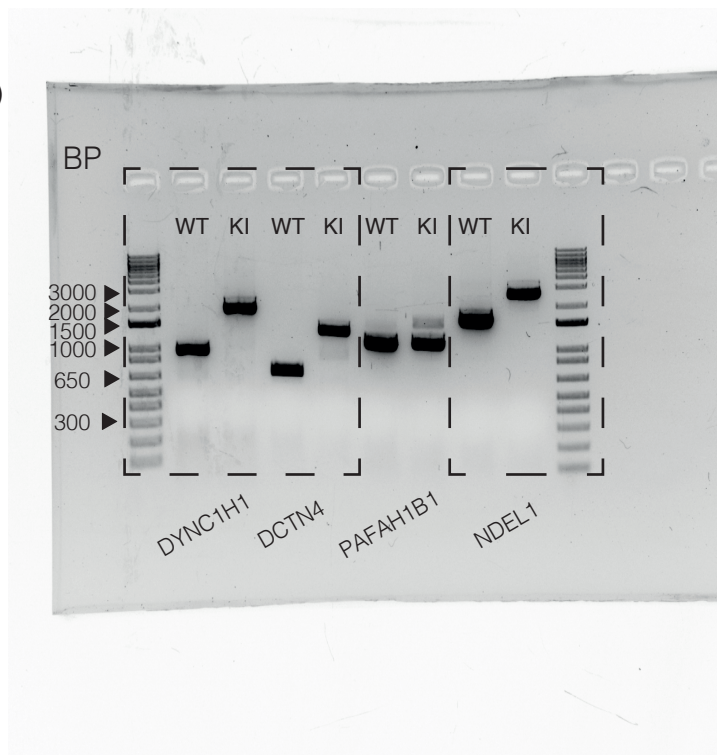

3SD)

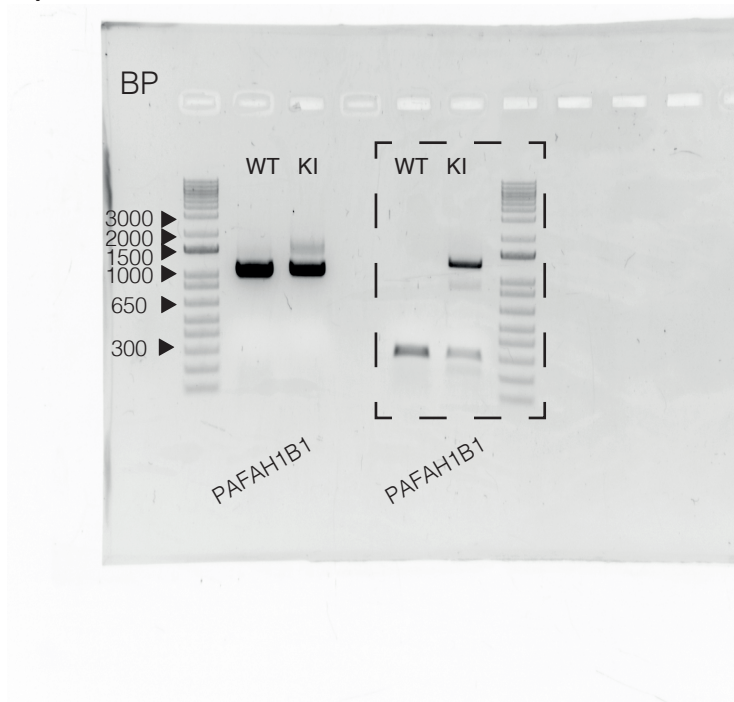

Supplement: SourceData FS3 — is the source file for Fig. S3. [file JCB_202309084_SourceDataFS3.pdf]
